# Room-temperature third-order nonlinear Hall effect in Weyl semimetal TaIrTe_4_

**DOI:** 10.1093/nsr/nwac020

**Published:** 2022-02-14

**Authors:** Cong Wang, Rui-Chun Xiao, Huiying Liu, Zhaowei Zhang, Shen Lai, Chao Zhu, Hongbing Cai, Naizhou Wang, Shengyao Chen, Ya Deng, Zheng Liu, Shengyuan A Yang, Wei-Bo Gao

**Affiliations:** College of Mathematics and Physics, Beijing University of Chemical Technology, Beijing100029, China; Division of Physics and Applied Physics, School of Physical and Mathematical Sciences, Nanyang Technological University, Singapore637371, Singapore; Institute of Physical Science and Information Technology, Anhui University, Hefei230601, China; Research Laboratory for Quantum Materials, Singapore University of Technology and Design, Singapore487372, Singapore; Division of Physics and Applied Physics, School of Physical and Mathematical Sciences, Nanyang Technological University, Singapore637371, Singapore; Division of Physics and Applied Physics, School of Physical and Mathematical Sciences, Nanyang Technological University, Singapore637371, Singapore; School of Materials Science and Engineering, Nanyang Technological University, Singapore639798, Singapore; Division of Physics and Applied Physics, School of Physical and Mathematical Sciences, Nanyang Technological University, Singapore637371, Singapore; Division of Physics and Applied Physics, School of Physical and Mathematical Sciences, Nanyang Technological University, Singapore637371, Singapore; CAS Center for Excellence in Nanoscience, National Center for Nanoscience and Technology, Beijing100190, China; School of Materials Science and Engineering, Nanyang Technological University, Singapore639798, Singapore; School of Materials Science and Engineering, Nanyang Technological University, Singapore639798, Singapore; Research Laboratory for Quantum Materials, Singapore University of Technology and Design, Singapore487372, Singapore; Division of Physics and Applied Physics, School of Physical and Mathematical Sciences, Nanyang Technological University, Singapore637371, Singapore; The Photonics Institute and Centre for Disruptive Photonic Technologies, Nanyang Technological University, Singapore637371, Singapore

**Keywords:** topological material, third-order nonlinear Hall effect, room temperature, Berry connection polarizability tensor

## Abstract

The second-order nonlinear Hall effect observed in the time-reversal symmetric system has not only shown abundant physical content, but also exhibited potential application prospects. Recently, a third-order nonlinear Hall effect has been observed in MoTe_2_ and WTe_2_. However, few-layer MoTe_2_ and WTe_2_ are usually unstable in air and the observed third-order nonlinear Hall effect can be measured only at low temperature, which hinders further investigation as well as potential application. Thus, exploring new air-stable material systems with a sizable third-order nonlinear Hall effect at room temperature is an urgent task. Here, in type-II Weyl semimetal TaIrTe_4_, we observed a pronounced third-order nonlinear Hall effect, which can exist at room temperature and remain stable for months. The third-order nonlinear Hall effect is connected to the Berry-connection polarizability tensor instead of the Berry curvature. The possible mechanism of the observation of the third-order nonlinear Hall effect in TaIrTe_4_ at room temperature has been discussed. Our findings will open an avenue towards exploring room-temperature nonlinear devices in new quantum materials.

## Teaser

In a type-II Weyl semimetal TaIrTe_4_, the pronounced 3^rd^ order nonlinear Hall effect signal has been observed, which can exist at room temperature and remain stable for months.

## INTRODUCTION

Recently, the nonlinear Hall effect that does not need the magnetic field has been observed in non-magnetic quantum materials. The frequency conversion metric is gradually becoming a hot research topic in condensed matter physics [1–6]. One important physical mechanism of the second-order nonlinear Hall effect is attributed to the Berry curvature dipole [1]. The Berry curvature and the Berry curvature dipole also help us to have a deeper understanding of several other physical phenomena, such as quantum Hall effect [7–10], orbital magnetization [11], gyrotropic Hall effect [12,13] and circular photogalvanic effect (CPGE) [14,15]. Besides, the Berry curvature dipole can introduce a Drude-like nonlinear optical process in non-centrosymmetric metal [16], and generate the nonlinear Nernst effect [17,18]. The nonlinear Hall effect has promising applications, such as high-frequency rectification [19], energy harvesting, wireless communications and infrared detectors [20], Berry curvature memory [21], electrical detection of ferroelectric-like metals [22] and two-dimensional piezoelectric-like devices [23]. Due to the large local Berry curvature and broken inversion symmetry, Weyl semimetals (WSMs) are expected to be ideal platforms to study the nonlinear Hall effect [24,25].

Beyond the second-order Hall effect, the higher-order nonlinear Hall effect is currently under active research, which will help us uncover new fundamental physical mechanisms and new applications. Particularly, the third-order Hall effect can dominate in non-magnetic centrosymmetric materials, where both linear and second-order Hall effects are suppressed. Recently, the third-order nonlinear Hall effect in MoTe_2_ and WTe_2_ was successfully detected [26]. It is further revealed that the physical mechanism of the third-order Hall effect is connected to the so-called Berry-connection polarizability (BCP) tensor [27,28], which is distinct from the second-order Hall effect. Thus, the third-order Hall effect offers a new characterization tool for a large class of materials and probes a new intrinsic band structure property.

However, it is noted that both MoTe_2_ and WTe_2_ are unstable in air, and the signals can only be detected at low temperatures, which severely limits their possible applications. Finding new material systems with air stability and sizable third-order Hall effect at room temperature is much desired. From the previous analysis [26,28], it is found that like Berry curvature, the BCP contribution is also pronounced around the band near degeneracies. Therefore, WSMs could be good candidates for exploring the sizable third-order Hall effect. As an important type-II WSM, TaIrTe_4_ [29,30] hosts the minimal number of Weyl points in all reported WSMs [31] and more importantly it is stable in air [32]. Therefore, exploring the third-order Hall effect in this material is meaningful for scientific research and practical application.

In this paper, we study the third-order nonlinear Hall effect of TaIrTe_4_ through transport measurements and symmetry analysis. We find two important characteristics of the third-order nonlinear Hall effect observed in TaIrTe_4_ compared to that observed in MoTe_2_ and WTe_2_ [26]: (i) the third-order nonlinear signals in TaIrTe_4_ are very robust and can stably exist for at least three months; (ii) the third-order nonlinear Hall effect in TaIrTe_4_ is pronounced and remains sizable and detectable at room temperature. Such room-temperature nonlinear Hall response has never been reported before. Our work will open an avenue towards building room-temperature third-order nonlinear Hall devices based on WSM systems.

## RESULTS AND DISCUSSION

### Results

**Figure 1.**
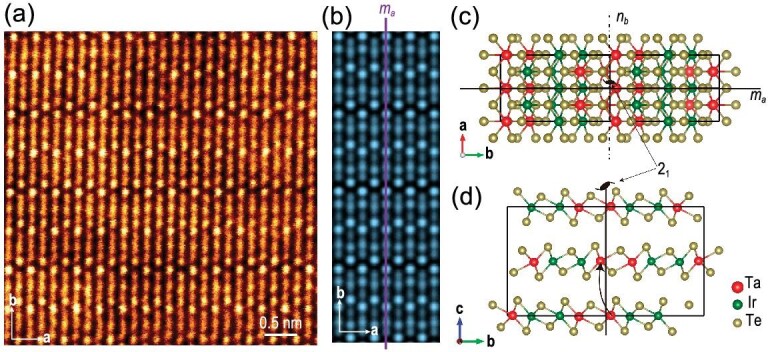
Crystal structure in TaIrTe_4_. (a) Experimental and (b) simulated TEM images of exfoliated TaIrTe_4_ flakes, with the a-b plane of the *T_d_* -phase crystal structure superimposed, respectively. A mirror plane *m_a_* of this structure is also shown in (b). (c) and (d) are the crystal structure of *T_d_*-TaIrTe_4_.

Before detecting the nonlinear Hall effect, we first studied the crystal structure and confirmed the ${T_d}$ structure of the TaIrTe_4_ crystal via transmission electron microscopy (TEM). TaIrTe_4_ samples for TEM measurements were prepared via the mechanical exfoliation method by using Scotch tape, then scanning transmission electron microscopy (STEM) was employed to study the microstructures of TaIrTe_4._ The atomic structure of TaIrTe_4_ was further confirmed by annular dark-field (ADF) STEM characterization. By comparing the intensity profile of the experimental ADF-STEM image (Fig. 1a) and simulated (Fig. 1b) STEM images, we found that the minimal periodic structure along the *b*-axis (highlighted by the rectangles in Fig. S1a–c respectively) exhibits identical intensities of each atomic column, confirming the ${T_d}$ phase of our TaIrTe_4_, which belongs to the space group *Pmn*2_1_. Each monolayer of TaIrTe_4_ consists of a layer of Ta (or Ir) atoms sandwiched between two layers of Te atoms in a distorted octahedral coordination, and multilayer TaIrTe_4_ is formed by stacking these monolayers with alternating layers rotated by 180^o^ (Fig. 1c and d), which includes a screw axis 2_1_ and a mirror plane *m_a_* perpendicular to this axis (Fig. 1c and d). The crystal symmetry is generated by two symmetry generators: a mirror plane *m_a_* and a glide mirror *n_b_*_._ The band structures of bulk TaIrTe_4_ with spin–orbit coupling were calculated, as shown in Fig. S1e.

**Figure 2.**
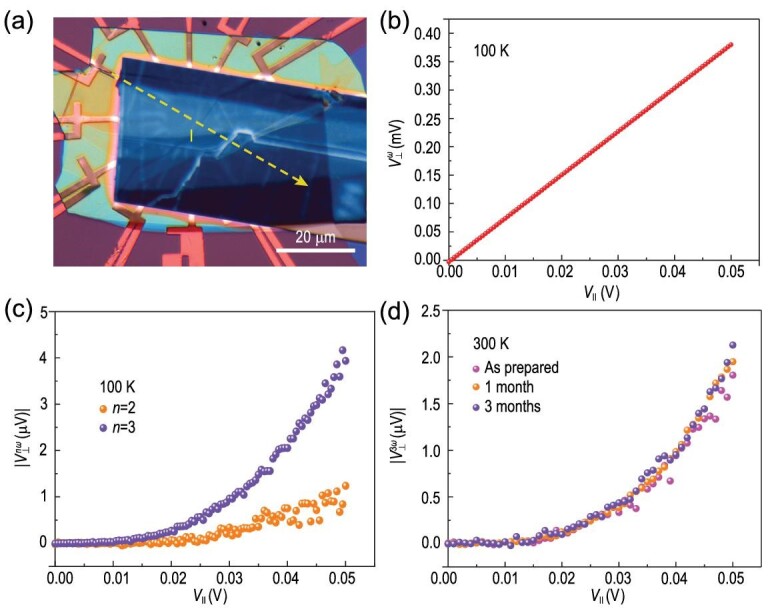
Nonlinear Hall effect in *T_d_-*TaIrTe_4_. (a) Optical image of a 12-electrode device with active areas protected by *h*BN. (b) First-harmonic ${V_ \bot }$ as a function of ${V_\parallel }$ at 100 K. (c) Second- and third-harmonic $V_ \bot ^{n\omega }$(*n* = 2, 3) as a function of ${V_\parallel }$ at 100 K. (d) Third-harmonic $V_ \bot ^{3\omega }\ $as a function of ${V_\parallel }$ measured at 300 K and the signals were taken from the as prepared and after one month or three months. All measured voltage signals at the transverse direction are perpendicular to the current direction, which is marked in (a).

Next, we constructed a device made of ${T_d}$-TaIrTe_4_ and explored its nonlinear Hall effect. To optimize the device for measuring Hall transport, a 161.2 nm thick ${T_d}$-TaIrTe_4_ flake (Fig. S2) with straight and long edges was transferred onto the circular disk device substrate with 12 Au/Cr electrodes, and the device was capped by hexagonal boron nitride (*h*BN) thin layers (Fig. 2a). Note that the 161.2 nm thick TaIrTe_4_ can be regarded as a bulk material. This point is important for our later analysis. By constantly applying the alternating current (ac) with fundamental frequency ω = 18.57 Hz to the device along the longitudinal direction under zero magnetic field (the current direction is labeled by the yellow line as shown in Fig. 2a), we measured the transverse voltage $V_ \bot ^{n\omega }$ (*n* = 1, 2, 3) directly by a lock-in amplifier (Zurich Instruments) in a phase-sensitive way at 100 K. We can see that the measured linear transverse voltage $V_ \bot ^\omega $ shows a linear relationship with increasing longitudinal voltage (${V_\parallel }$) and keeps a very small value ∼1% of ${V_\parallel }$ (Fig. 2b). The linear Hall response should vanish due to the preserved time-reversal symmetry, and the finite value of $V_ \bot ^\omega $ (∼1% of the ${V_\parallel }$) observed here is coming from the anisotropy of the crystal structure (hence the resistivity tensor) and the misalignment of the electrodes with the crystal axes of *T_d_*-TaIrTe_4_ [3], rather than the misalignment between the Hall contacts (Fig. S3). Importantly, along the transverse direction, we can observe not only a second-order voltage $V_ \bot ^{2\omega }$, but also a third-order voltage $V_ \bot ^{3\omega }$ (the magnitudes of $V_ \bot ^{2\omega }$ and $V_ \bot ^{3\omega }$ are ∼0.1% of ${V_\parallel }$) (Fig. 2c), and $V_ \bot ^{2\omega }$ and $V_ \bot ^{3\omega }$ exhibit clear quadratic and cubic relationships with the longitudinal voltage ${V_\parallel }$, respectively. Importantly, the third-order voltage $V_ \bot ^{3\omega }$ is almost four times larger than that of the second-order.

The suppression of the second-order Hall response is due to the crystal symmetry. As discussed above, although bulk ${T_d}$-TaIrTe_4_ breaks the inversion symmetry, it has a C_2z_ symmetry in its point group, originated from the 2_1_ screw axis along the out-of-plane direction (which connects the atoms in two neighboring layers, see Fig. 1c and d). For the *in-plane* second-order nonlinear response:

(1) \begin{equation*}{\rm{\ }}j_\alpha ^{\left( {2\omega } \right)} = \chi _{\alpha \beta \gamma }^{2\omega }\ {E_\beta }{E_\gamma },\end{equation*}

where $\alpha ,\ \beta ,\ \gamma \in \{ {x,\ y} \}$ in our case, and $\chi _{\alpha \beta \gamma }^{2\omega }$ is the second-order nonlinear conductivity. Under C_2z_, the current and the *E* field would reverse, which suppresses the second-order *in-plane* response in the bulk of the material. This explains why the second-order signal is weak in our measurement. One also noted that the screw axis 2_1_ is broken at the surfaces of the sample, so the second-order Hall signal we measured should be mainly from the surfaces. Actually, a recent work, ref. [20], studied the relationship between the thickness of TaIrTe_4_ and the second-order Hall effect, and found enhanced second-order Hall effect in few-layer TaIrTe_4_. The result is consistent with our analysis here.

Now, let us turn to the third-order nonlinear Hall effect. The value of the third-ordered nonlinear Hall signal at ${V_\parallel }$= 0.05 V is four times larger than that of the second-order Hall signal (Fig. 2c). In the general form, the third-order current response can be expressed as

(2) \begin{equation*}{\rm{\ }}j_\alpha ^{\left( {3\omega } \right)} = \chi _{\alpha \beta \gamma \lambda }^{3\omega }\ {E_\beta }{E_\gamma }{E_\lambda },\end{equation*}

where $\chi _{\alpha \beta \gamma }^{3\omega }$ is the third-order nonlinear conductivity. One notes that, distinct from the second order, the C_2z_ symmetry does not forbid the third-order nonlinear Hall effect.

Importantly, the third-order nonlinear Hall effect here is detectable at room temperature (300 K); the material is very stable in air, [32] and the measured third-order nonlinear Hall signal can persist after three months (Fig. 2d). Note that our device fabrication was performed under ambient conditions. We did not observe any degradation of the sample in the process. It is worth noting that the main purpose of encapsulating *h*BN in our device is to improve the electrical contact between TaIrTe_4_ and Cr/Au electrodes at low temperature. Our device is fabricated by transferring TaIrTe_4_ onto prepatterned electrodes; with *h*BN covering on the sample, good electric contact can be achieved. This is in contrast to WTe_2_ or MoTe_2_, where the fabrication has to be done in a vacuum or in inert gas environments and *h*BN encapsulation is required for protecting these materials [33]. Our conclusion is that the third-order nonlinear Hall effect observed in air-stable TaIrTe_4_ is very robust, which could pave the way to practical application.

**Figure 3.**
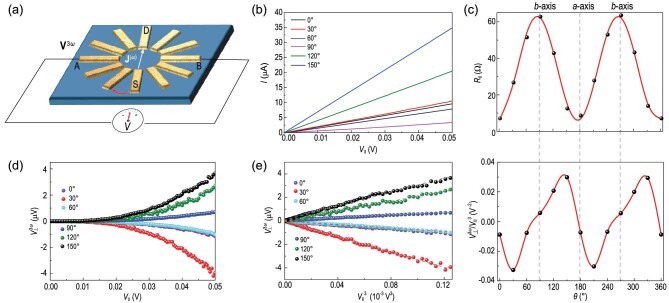
Angular dependence of the nonlinear Hall effect in *T_d_*-TaIrTe_4_. (a) Electrode geometry for angle-dependent measurements. The driving field is applied between two opposite electrodes, and the voltage drop is measured between the other two opposite electrodes at the transverse direction. By rotating the measurement framework in a clockwise direction as shown by the red arrow, signals of different lattice directions are recorded. (b) First-harmonic *I*-*V* curves for different directions in TaIrTe_4_. (c) The upper panel is ${R_\parallel }$ as a function of $\theta $, the black dots are experimental data and the line is fitted to the experimental data. The lower panel is the ${V_{\bot}^{3\omega }}/{V_\parallel^{3} }$ as a function of $\theta $, the black dots are experimental data and the red line is the spline of each experimental data set. (d) ${V_{\bot}^{3\omega }}$ depends nonlinearly on the first-harmonic ${V_\parallel }$ for different directions. (e) ${V_{\bot}^{3\omega }}$ depends linearly on the cubic of the first-harmonic ${V_\parallel }$ for different directions. All the tests are implemented at 100 K.

To further characterize this third-order nonlinear Hall effect, we studied the angle dependence of the third-order signal as a function of current direction at 100 K. As shown in Fig. 3a, the fundamental frequency (ω = 18.57 Hz) ac current was applied to the device through a one source electrode (S) to the opposite drain electrode (D). At the same time, we measured the voltage drop between another pair of electrodes at the transverse direction, labeled as A and B, which is the transverse voltage. *θ* labels the current direction and it is measured from electrode #1 as 0° clockwise (Fig. S3). The angle between two neighboring electrodes is 30°. Signals in different lattice directions were recorded by rotating the measurement frame while keeping the relative positions (S-A-D-B in clockwise order) unchanged. It is worth noting that in the transverse voltage measurement, when we changed the source (S) and drain (D) directions, A and B were also switched to make sure the relative positions (S-A-D-B in clockwise order) were maintained, as shown in Fig. S4. The longitudinal current $I_\parallel ^\omega $ shows a good linear relationship with longitudinal voltage ${V_\parallel }$ at all angles (Fig. 3b). The angle dependence of ${R_\parallel }$ is presented in Fig. 3c (upper panel) as a function of θ. The longitudinal resistances ${R_\parallel }$ can be expressed as:

(3) \begin{equation*}{R_{||}}\!\left( \theta \right){\rm{ }} = {R_b}{\cos ^2}\theta + {R_a}{\sin ^2}\theta, \end{equation*}

where *R_a_* and *R_b_* (>*R_a_*) are the resistances along the *a*- and *b*-axis, respectively. With this function, we fitted (the red line shown in the upper panels in Fig. 3c) the angle dependence of ${R_\parallel }$ and found a good agreement with a 2-fold angular dependence. The resistance anisotropy *r* defined as *R_a_*/*R_b_* is ∼0.1. After fixing the crystal axis orientations, we focused on the third-order signal. As discussed above, the third-order nonlinear transverse voltage $V_ \bot ^{3\omega }$ shows a cubic relationship with longitudinal voltage ${V_\parallel }$ (Fig. 3e). It also exhibits a 2-fold angular dependence, as shown in Fig. 3e. At the same time, $V_ \bot ^{3\omega }$ vanishes at θ ∼0°, 90°, 180°, 270° and 360°, which corresponds to the crystal *a*- or *b*-axis (lower panel in Fig. 3c). The angular dependence of the third-order nonlinear response can be well fitted by the formula derived for the *Pmn2_1_* symmetry in ref. [26] (also see Fig. S4). The vanishing $V_ \bot ^{3\omega }$ at specific angles can be readily understood from the following analysis. When the driving current is along the *a*-axis and the voltage is measured along the *b*-axis, $\beta ,\ \gamma ,\ \lambda \ = \ x$ and $\alpha \ = \ y$ accordingly, then under the *m_a_* symmetry ${E_x}$($j_y^{( {3\omega } )}$) is reversed (invariant), which leads to the vanishing $V_ \bot ^{3\omega }$ according to Equation (2). Similarly, the *n_b_* symmetry also forces the signal to vanish when the driving current is along the *b*-axis.

### Discussion

According to the conventional semi-classical equations of motion [34], the third-order nonlinear Hall current induced by the Berry curvature can be written as:

(4) \begin{eqnarray*} &&{{\boldsymbol{j}}^{\left( {3,{\rm{Berry}}} \right)}} \nonumber\\ &&\quad = - e\ \int \left[ {d{{\bf k}}} \right]\!e\!{{\bf E}} \times {{\boldsymbol \Omega }}{f^{\left( 2 \right)}} \nonumber\\ &&\quad = {-} \frac{{{e^4}{\tau ^2}}}{{{\hbar ^2}}}\int \left[ {d{{\bf k}}} \right]\!{{\bf E}}\! \times\! {{\boldsymbol \Omega }}\!\left( {{\bf k}} \right){({{\bf E}}\! \cdot\! {\nabla _{{\bf k}}})^2}{f_0}\!\left( {{\bf k}} \right),\nonumber\\ \end{eqnarray*}

where τ is the relaxation time of carriers and ${f_0}( {{\bf k}} )$ is the Fermi distribution. The term ${{\boldsymbol \Omega }}( {{\bf k}} ){({{\bf E}} \cdot {\nabla _{{\bf k}}})^2}{f_0}( {{\bf k}} )$ may be called the Berry curvature quadrupole. However, because ${{\boldsymbol \Omega }}( {{\bf k}} )$ is odd under time reversal [${{\boldsymbol \Omega }}( {{\bf k}} )\ = \ - {{\boldsymbol \Omega }}( { - {{\bf k}}} )$], while $\nabla _{{\bf k}}^2{f_0}( {{\bf k}} )$ is even, one finds that Equation (4) vanishes identically for time-reversal invariant systems. In other words, unlike the second-order nonlinear Hall effect, the *intrinsic* Berry curvature will not contribute to the third-order Hall effect in non-magnetic systems [35].

As shown in refs [26] and [28], the third-order Hall effect has a different origin with the second-order Hall effect, and the former originates from the BCP of the band structure. BCP arises when studying the field corrections to the Berry curvature. Under an applied *E* field, there is an induced Berry curvature ${{{\boldsymbol \Omega }}^E}$ due to the interband mixing [27]:

(5) \begin{equation*}{\rm{\ }}{{{\boldsymbol \Omega }}^E} = {\nabla _{{\bf k}}}\ \times \left( {{{\bf GE}}} \right),\end{equation*}

where

(6) \begin{equation*}{{\rm{G}}_{ij}} = \ 2{\mathop{\rm Re}\nolimits} \mathop \sum \limits_{n \ne 0} \frac{{{{\left( {{V_i}} \right)}_{0n}}{{\left( {{V_j}} \right)}_{n0}}}}{{{{\left( {{\varepsilon _0} - {\varepsilon _n}} \right)}^3}}},\end{equation*}

here **G** is the BCP tensor, ϵ_0_ and ϵ*_n_* are band energies for bands 0 and *n*, respectively, *i* and *j* refer to the spatial components, and (*V_i_*)*_0n_* is the interband matrix element of the velocity operator. It is important to note that BCP has symmetry characters that are distinct from the intrinsic Berry curvature. The intrinsic Berry curvature **Ω** is odd under time reversal and is even under inversion. In comparison, BCP is even under both time reversal and inversion, according to Equation (6). As a result, it can exist in systems that preserve both time reversal and inversion symmetries, and contribute to the third-order Hall effect. Table 1 summarizes the differences between Berry curvature and BCP.

**Table 1.** Comparison of the symmetry properties of Berry curvature and BCP.

		Berry curvature	BCP
			
Symmetry operation	Time reversal	Odd	Even
	Inversion	Even	Even

Based on the extended semi-classical theory by Gao, Yang and Niu [27,36], the following contribution for the third-order current response has been derived [26,28], which is linear in the relaxation time:

(7) \begin{eqnarray*}{\rm{\ }}{{\boldsymbol{j}}^{\left( 3 \right)}} = \ - \tau \frac{{{e^3}}}{{{\hbar ^2}}}\int \left[ {d{{\bf k}}} \right]\left\{ {\left[ {{{\bf E}} \times \left( {{\nabla _{{\bf k}}} \times {{\bf GE}}} \right) - {\nabla _{{\bf k}}}\left( {{{\bf E}} \cdot {{\bf GE}}} \right)} \right]\left( {{{\bf E}} \cdot {\nabla _{{\bf k}}}} \right){f_0} - \frac{\hbar }{2}{{{\bf v}}_0}\left( {{{\bf E}} \cdot {{\bf GE}}} \right)\left( {{{\bf E}} \cdot {\nabla _{{\bf k}}}} \right)f_0^{\prime}} \right\}.\nonumber\\\end{eqnarray*}

Meanwhile, the conventional field driving of electrons in non-magnetic materials also gives a contribution proportional to ${\tau ^3}$ [28], which may be called the Drude-like term:

(8) \begin{eqnarray*} {{\boldsymbol{j}}^{\left( {3,{\rm{Drude}}} \right)}} &=& - e\ \int \left[ {d{{\bf k}}} \right]\frac{{\partial \tilde{\varepsilon }}}{{\hbar \partial {{\bf k}}}}{f^{\left( 3 \right)}} \nonumber\\ &=& - \frac{{{e^3}{\tau ^3}}}{{{\hbar ^3}}}\int \left[ {d{{\bf k}}} \right]\frac{{\partial \tilde{\varepsilon }}}{{\hbar \partial {{\bf k}}}}{({{\bf E}} \cdot {\nabla _{{\bf k}}})^3}{f_0}.\nonumber\\ \end{eqnarray*}

**Figure 4.**
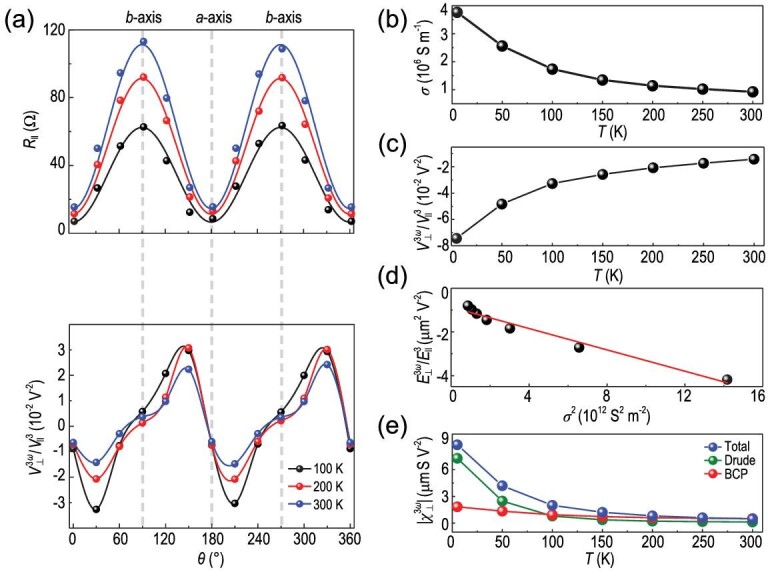
Temperature dependence of the nonlinear Hall effect in *T_d_* -TaIrTe_4_. (a) The lower panel is the longitudinal resistance ${R_\parallel }$ as a function of $\theta $ at different temperatures from 100 to 300 K. The dots are experimental data, and the lines are fitted to the experimental data. The upper panel is the $V_ \bot ^{3\omega }$/$V_\parallel ^3$ as a function of $\theta $ with different temperatures from 100 to 300 K, the dots are experimental data and the lines are the splines of each experimental data set. (b) The longitudinal conductivity σ as the function of temperature. (c) The third nonlinear Hall effect $V_ \bot ^{3\omega }$/$V_\parallel ^3$ as the function of temperature. (d) $\frac{{| {E_ \bot ^{3\omega }} |}}{{E_\parallel ^3}}$ with the square of longitudinal conductivity *σ*. (e) ${\rm{\chi }}_ \bot ^{3\omega }$ including the Drude-like and BCP-like parts as the function of temperature. For (b), (c) and (d), the signal was taken from the data when the driving current was applied at 30 degree. For (e), the signal was taken from the data when the driving current was applied at 120 degree.

These two contributions can be separated from a scaling analysis. The temperature dependence of the material's conductivity and the third-order nonlinear response $V_ \bot ^{3\omega }$ are shown in Fig. 4. Figure 4a (upper panel) shows the angular dependence of the longitudinal resistances ${R_\parallel }$ (dots) at different temperatures. We can see that the longitudinal resistance ${R_\parallel }$ increases with temperature. Figure 4a (lower panel) summarizes the angular dependence $V_ \bot ^{3\omega }$/$V_\parallel ^3$ at different temperatures as a function of $\theta $, and the third-order nonlinear response $V_ \bot ^{3\omega }$/$V_\parallel ^3$ decreases when the temperature increases from 100 to 300 K. At the same time, the material's longitudinal conductivity σ decreases as the temperature increases from 5 K to 300 K, as shown in Fig. 4b. Next, we focus on the third-order nonlinear response $V_ \bot ^{3\omega }$ from 5 K to 300 K. As observed at 100 K, $V_ \bot ^{3\omega }$ depends linearly on $V_\parallel ^3$ at all temperatures (Fig. S4) and the slope of $V_ \bot ^{3\omega }$ versus $V_\parallel ^3$ ($V_ \bot ^{3\omega }/V_\parallel ^3$) in TaIrTe_4_ decreases with temperature (Fig. 4c). We plot $\frac{{| {E_ \bot ^{3\omega }} |}}{{E_\parallel ^3}}$ versus σ^2^ as shown in Fig. 4d, which confirms the scaling relation that

(9) \begin{equation*}{\rm{\ }}\frac{{\left| {E_ \bot ^{3\omega }} \right|}}{{E_\parallel ^3}} = \ \xi {\sigma ^2} + \eta ,\end{equation*}

where $\xi $ and $\eta $ are constants, and ${E^{3\omega }} = {j^{3\omega }}/\ \sigma $. The left-hand side can be rewritten as $\frac{{| {E_ \bot ^{3\omega }} |}}{{E_{||}^3}} \approx \frac{{{\chi ^{3\omega }}}}{\sigma }$. Since the longitudinal conductivity $\sigma \ $depends linearly on the scattering time $\tau $, Equation (9) shows that third-order response coefficient ($\chi _ \bot ^{3\omega }$) has two contributions that scale with $\tau $ and ${\tau ^3}$, which are consistent with our above analysis. The contribution of the third-order nonlinear coefficient can be divided into the Drude-like and BCP-like parts as shown in Fig. 4e. The fitted parameters for TaIrTe_4_ are presented in Table S1 in the $\rule{16pc}{0.5pt} $ supplementary data, and are compared with the results for MoTe_2_ in ref. [26]. It was found that the BCP-like contribution (parameter $\eta $) in TaIrTe_4_ is more than two times that of MoTe_2_, and the Drude-like contribution in TaIrTe_4_ is also much larger than MoTe_2_. Since the BCP-like contribution decays with temperature much slower than the Drude-like contribution, the larger BCP-like contribution in TaIrTe_4_ plays a crucial role in the pronounced third-order Hall effect that remains detectable at room temperature. A detailed analysis of the temperature dependence is presented in Figs S6–S8.

Finally, as discussed, the second-order response is suppressed here by the 2_1_ screw axis in the bulk material, and the remaining signal should be from the surfaces where the screw axis is broken. If one wishes to further suppress the signal, one possible way is to choose a material with a C_2z_ axis instead of a screw. Meanwhile, increasing the sample thickness can also decrease the relative weight of the second-order signal in TaIrTe_4_.

### CONCLUSION

In conclusion, in type-II WSM TaIrTe_4_, we have revealed a room-temperature third-order nonlinear Hall effect, and found that the third-order signal in TaIrTe_4_ can persist for at least three months and keep almost the same value at 300 K. The BCP tensor plays an important role in the third-order Hall effect, which is different from both linear and second-order nonlinear Hall effects. These findings will deepen our understanding of the BCP tensor, which is an intrinsic band geometric quantity and plays an important role in the nonlinear phenomena. This work paves the way for possible room-temperature applications based on the third-order nonlinear Hall effect in WSMs.

## METHODS

### Crystal preparation

TaIrTe_4_ single crystals were prepared by solid-state reaction using tellurium as flux. Tellurium pieces (99.999%), tantalum powder (99.99%) and iridium powder (99.999%) were purchased from Sigma-Aldrich and were loaded (at an atomic ratio of Te:Ta : Ir = 12 : 1 : 1) into a quartz tube, which was flame sealed under a high vacuum of 10^−6^ torr. The quartz tube was placed in the tube furnace, slowly heated to 1000°C and left there for 100 h, before being allowed to cool to 600°C at a rate of 0.8°C h^–1^, then cool further to room temperature. At the end, shiny and needle-like TaIrTe_4_ single crystals were obtained from the product.

### Device fabrication

The TaIrTe_4_ flakes were mechanically exfoliated from the bulk crystal onto the polydimethylsiloxane and then released onto the SiO_2_/Si substrate with Cr/Au electrodes. This was followed by the stacking of h-BN on the TaIrTe_4_. The TaIrTe_4_ flakes were identified by optical microscopy, and their thickness was measured by an atomic force microscope (AFM). The crystal orientation of TaIrTe_4_ flakes was first estimated from the flake shape and then determined by angle-dependent measurements.

### Electrical measurements

An alternating electric field was applied to the sample, and the voltage drop was recorded by a phase-sensitive lock-in amplifier. The measurement of the angle dependence was performed by rotating the reference frame.

## Supplementary Material

nwac020_Supplemental_File
