# Supplementary material for: Room-temperature third-order nonlinear Hall effect in Weyl semimetal TaIrTe4
Source: Natl Sci Rev. 2022 Feb 14;9(12):nwac020. doi: 10.1093/nsr/nwac020 (PMC9869080; doi:10.1093/nsr/nwac020)
Supplement: nwac020_Supplemental_File [file nwac020_supplemental_file.docx]

**Supplementary Information**
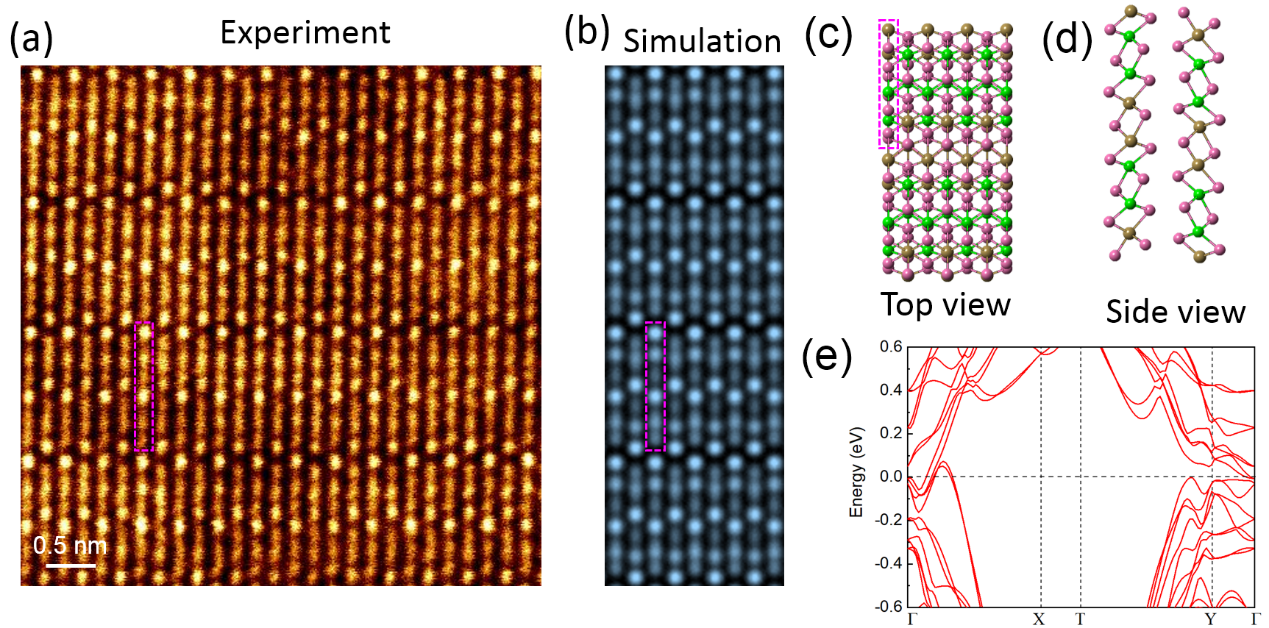


Figure S1. **TEM and Band structure of TaIrTe_4_. (a), (b),** Experiment and simulation TEM image of one of our exfoliated TaIrTe_4_ flakes, with the 𝑎−b plane of the T*_d_* phase crystal structure superimposed, respectively. The only mirror plane of this structure (*m_a_*) is also shown in **(b)**. **(c)**, **(d),** The top and side view of the T*_d_* phase crystal structure of TaIrTe_4_. The only mirror plane of this structure (*m_a_*) is also shown in **(c).** (e) Band structure of TaIrTe_4_ with spin–orbit coupling.

**Density-functional theory (DFT) Calculation of the band structure of TaIrTe_4_:**

The first-principle calculations of TaIrTe_4_ crystal were carried out by the VASP package based on the density functional theory (DFT) ^[1]^. For the structural relaxation and electronic structures calculations, the kinetic energy cutoff is set to 500 eV. The *k*-point meshes 12×6×6 are used to optimize the geometric structures of TaIrTe_4_ crystal. The stress force and energy convergence criterions are chosen as 0.01 eV/Å and 10^-5^ eV, respectively. The van der Waals interaction force is treated by a semi-empirical DFT-D3 method ^[2, 3]^. The relaxed lattice parameters of TaIrTe_4_ crystal are *a*=3.77 Å, *b*=12.42 Å, and *c*=13.18 Å ^[4]^. The SOC effects are included in the selfconsistent calculations of electronic structure.


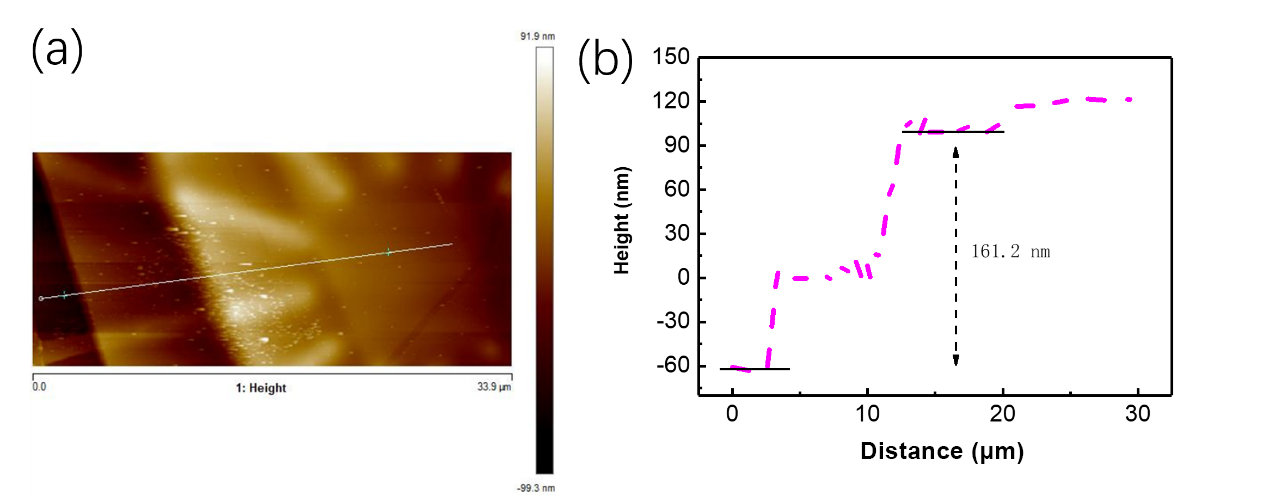


Figure S2. **Atomic Force Microscope** (**AFM) measurement of the TaIrTe_4_ device.** The thickness of TaIrTe_4_ is 161.2 nm.


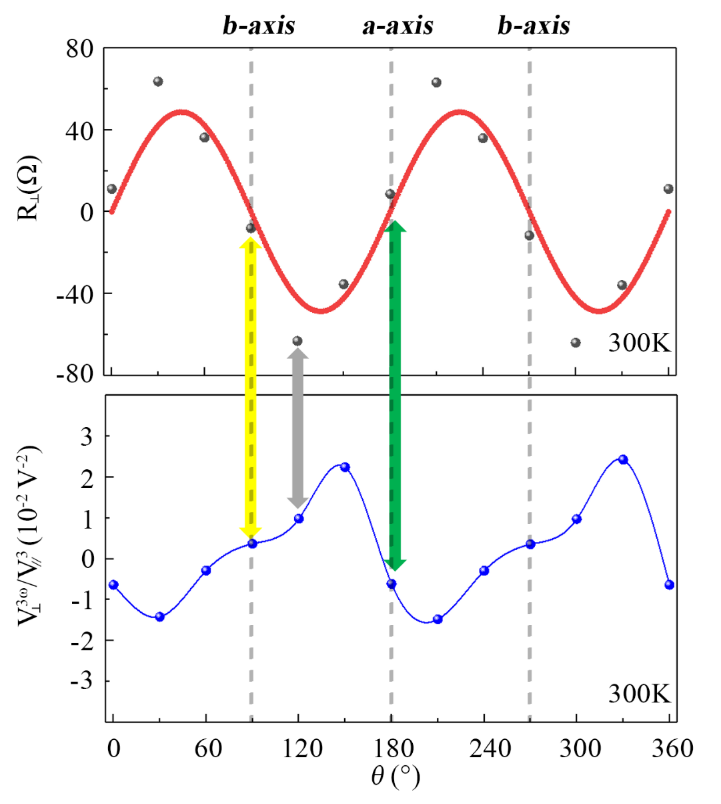


Figure S3. (**Upper panel**) $R_{\perp}$ ($=V_{\perp}/I_{//}$) and (**lower panel**) $V_{\perp}^{3\omega}/V_{//}^{3}$ as a function of θ in the *T_d_*-TaIrTe_4_ device of Figure 4a in the main text.

We can see from Figure 2 (b) of the main text that the measured linear transverse voltage $V_{\perp}^{\omega}$ shows a linear relationship with increasing longitudinal voltage (V_//_) and keeps a very small value ~1% of the V_//_. Such small $V_{\perp}^{\omega}$ is dominated by the intrinsic resistance anisotropy of Td-TaIrTe_4_, not the misalignment between the Hall contacts. If $V_{\perp}$ or ($R_{\perp}=V_{\perp}/I_{//}$) is dominated by misalignment, its angle dependence should not follow the anisotropic resistance model of $R_{\perp}$. However, as shown in the Figure S3, the measured $R_{\perp}$at 300 K can be fitted well with the anisotropic resistance model:${(R}_{b}-R_{a})sin\theta cos\theta$, which is consistent with the previous reports as well. We can further exclude the influence of $R_{\perp}$on 3^rd^ harmonic signal by comparing their angle dependence. As indicated by the gray arrow in Fig. S3, when $R_{\perp}$ reaches the maximum value, the corresponding $V_{\perp}^{3\omega}/V_{//}^{3}$ shows a value close to minimum (zero). And as indicated by the yellow arrow, when $R_{\perp}$ value becomes smaller compared to the case of gray arrow, the corresponding shows a much larger value. Furthermore, $R_{\perp}$ indicated by yellow and green arrows shows similar values, but the corresponding $V_{\perp}^{3\omega}/V_{//}^{3}$ values are very different. Thus, the finite value of $V_{\perp}^{\omega}$ (~1% of the V_//_) is not coming from the misalignment between the Hall contacts (Figure S3)*.*


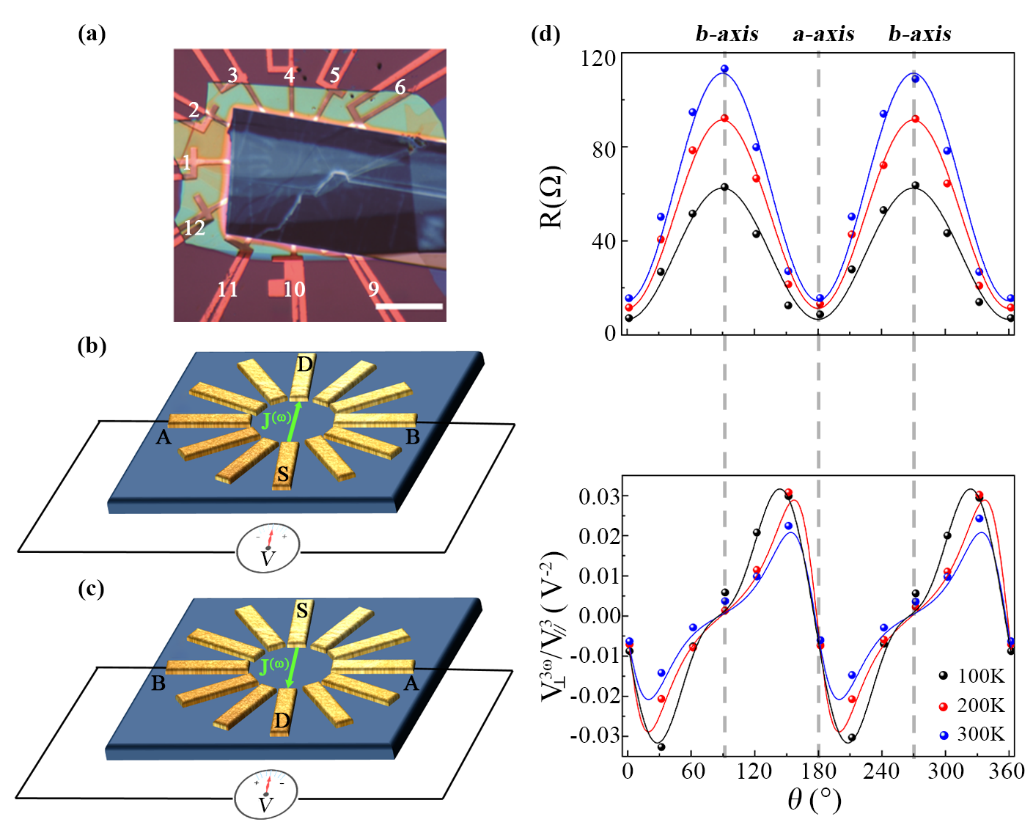


Figure S4. **(a)**, Optical image of our TaIrTe_4_ device, with the electrodes numbered. **(b)-(c),** Electrode geometry for angle-dependent measurements. The driving field is applied between two opposite electrodes, and the voltage drop is measured between the other two opposite electrodes at the transverse direction. By rotating the measurement framework along the clockwise direction shown as the red arrow, signals of different lattice directions are recorded. In the transverse (Hall) voltage measurement, when we changed the source (S) and drain (D) direction, A and B were also changed to make sure the relative positions (S-A-D-B in clockwise order) keeping unchanged. **(d),** The upper panel is the longitudinal conductivity $R_{\parallel}$ as a function of $\theta$ at different temperatures from 100 to 300 K. The symbols are experimental data, and the lines are fitted to the experimental data. The lower panel is the slop of $V_{\perp}^{3\omega}$/$V_{\parallel}^{3}$ as a function of $\theta$ with different temperatures from 100 to 300 K, the symbols are experimental data, and the lines are fitted to the experimental data.


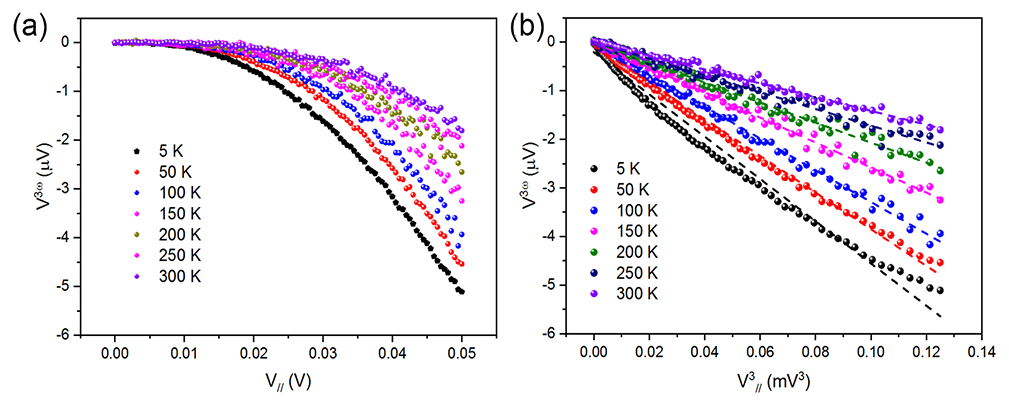


Figure S5. **(a)**, The 3^rd^ nonlinear Hall effect at temperatures ranging from 5 to 100 K (5, 50, 100, 150, 200, 250, and 300 K). **(b)**,$V_{\perp}^{3\omega}$ depends linearly on $V_{\parallel}^{3}$ at temperatures ranging from 5 to 100 K (5, 50, 100, 150, 200, 250, and 300 K). The symbols are experimental data and the dashed line in b is a linear fit to the experimental data. **(a)** and **(b)** are taken from the same device of 30 degree’s electrodes as in Figure 2**a**.


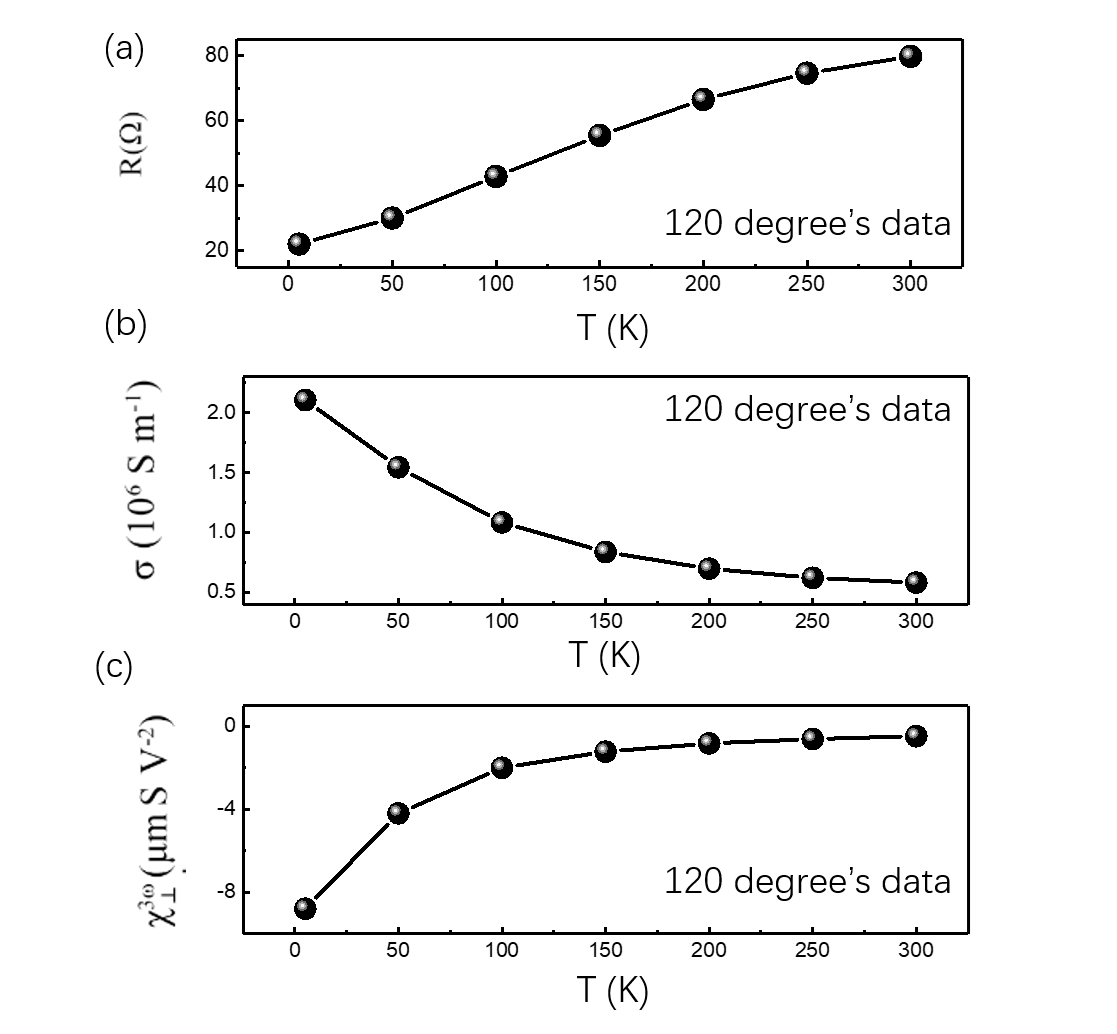


Figure S6. **(a),** $R_{\parallel}$ as a function of temperature. The conductivity σ **(b),** and the 3^rd^ order nonlinear Hall conductivity $\chi_{\perp}^{3\omega}$ **(c),** as a function of temperature. The signal is taken from the 120 degree’s data.

To express the scaling relation in terms of intensive quantities such as the electric field strengths and the conductivity, we use the relationship $E_{\perp}^{3\omega}=\frac{V_{\perp}^{3\omega}}{L_{\perp}}$ , $E_{\parallel}=\frac{V_{\parallel}}{L_{\parallel}}$ _,_ $\frac{E_{\perp}^{3\omega}}{E_{\parallel}^{3}}=\frac{V_{\perp}^{3\omega}}{L_{\perp}}\left( \frac{L_{\parallel}}{V_{\parallel}} \right)^{3}=\frac{V_{\perp}^{3\omega}}{V_{\parallel}^{3}} \frac{L_{\parallel}^{3}}{L_{\perp}}$ _,_$\sigma=\frac{j}{E}=\frac{I}{S}\cdot\frac{L}{V}=G\frac{L}{S}$ (for the 120 degree conductivity), where L is the length between two opposite electrodes and S is the cross-sectional area of the channel. We can get the values of 3^rd^ order nonlinear Hall conductance $\chi_{\perp}^{3\omega}$ of 120 degree in TaIrTe_4_ from $\chi_{\perp}^{3\omega}=\frac{j_{\perp}^{3\omega}}{E_{\parallel}^{3}}=\frac{j_{\perp}^{3\omega}}{E_{\perp}^{3\omega}}\frac{E_{\perp}^{3\omega}}{E_{\parallel}^{3}}=\sigma_{\perp}\frac{E_{\perp}^{3\omega}}{E_{\parallel}^{3}}=G_{\perp}\frac{L_{\perp}}{S} \frac{E_{\perp}^{3\omega}}{E_{\parallel}^{3}}$_._

**Temperature dependence of the nonlinear Hall coefficients**

The 3^rd^ order nonlinear Hall effect coefficient $\chi_{\perp}^{3\omega}$ of MoTe_2_ and TaIrTe_4_ under different temperature was shown in Fig. S7, from which we can see that the 3^rd^ order nonlinear Hall effect coefficient $\chi_{\perp}^{3\omega}$ of TaIrTe_4_ is bigger than that of MoTe_2_ at low temperature (5-100 K), but it decays slower with temperature increasing than that of MoTe_2_. At the same time, as shown in Fig. S7 (b) that the slop of $V_{\perp}^{3\omega}/V_{//}^{3}$ of TaIrTe_4_ was also bigger and decayed more slowly than that of MoTe_2_, which means that, the 3^rd^ order Hall signal in TaIrTe_4_ is more significant than that of MoTe_2_ when applying the same driving voltage.


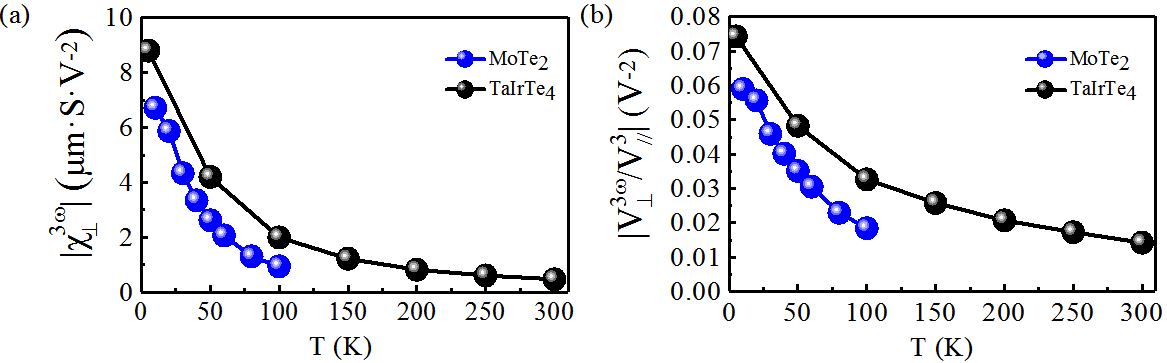


Figure S7. **(a)** the absolute value of the 3^rd^ order nonlinear Hall effect coefficient $\chi_{\perp}^{3\omega}$ and **(b)** $V_{\perp}^{3\omega}$/ $V_{\parallel}^{3}$ as a function of temperature of MoTe_2_ and TaIrTe_4_, respectively.

In order to explain the temperature dependence of the TNHE of two materials, we divide the 3^rd^ Hall effect into the Drude-like and BCP-like parts. According to Eq. (9) of the manuscript,

$$\frac{\left| E_{\perp}^{3\omega} \right|}{E_{\parallel}^{3}}=\xi\sigma^{2}+\eta,$$

where $E^{3\omega}=j^{3\omega}/\sigma$. According to Eq. (7) and Eq. (8) of the main text,

$\left\{ \begin{aligned} &J_{BCP}^{(3\omega)}\propto\tau\propto\sigma, \\ &J_{Drude}^{(3\omega)}\propto\tau^{3}\propto\sigma^{3}, \end{aligned} \right.$ S

we will get that

$J_{\perp}^{3\omega}=\left( \xi\sigma^{3}+\eta\sigma\right)E_{\parallel}^{3}=\left( \chi_{int rsic}^{3\omega}+\chi_{Drude}^{3\omega} \right)E_{\parallel}^{3},$ S

and

$\left\{ \begin{aligned} &\chi_{Drude}^{3\omega}=\xi\sigma^{3}, \\ &\chi_{intrsic}^{3\omega}=\eta\sigma. \end{aligned} \right.$ S

Table S1 Fitting parameters of MoTe2 and TaIrTe_4_

|  | $\xi$ (μm^4^/A^2^) | $\eta$ (μm^2^V^-2^) |
| --- | --- | --- |
| MoTe_2_ | 0.030 | 0.36 |
| TaIrTe_4_ | -0.244 | -0.87 |

The fitting parameters of MoTe_2_ and TaIrTe_4_ are shown in Table S1. According to Eq. S(3) and Table S1, the contribution of the 3^rd^ order nonlinear Hall effect coefficient ($\chi_{\perp}^{3\omega}$) are divided by the Drude-like and BCP-like parts as shown in Fig. S8. For the Drude-like term, it decays more significant with temperature [Eq. S(1) and S(3)]. For TaIrTe_4_, the Drude term are smaller than that of MoTe_2_, and it decay nearly to zero at high temperature. Besides, TaIrTe_4_ has higher composition of BCP-like term, it decays slowly with temperature, and dominants at high temperature [Fig. S2], so 3^rd^ Hall effect of TaIrTe_4_ can survive at room temperature.


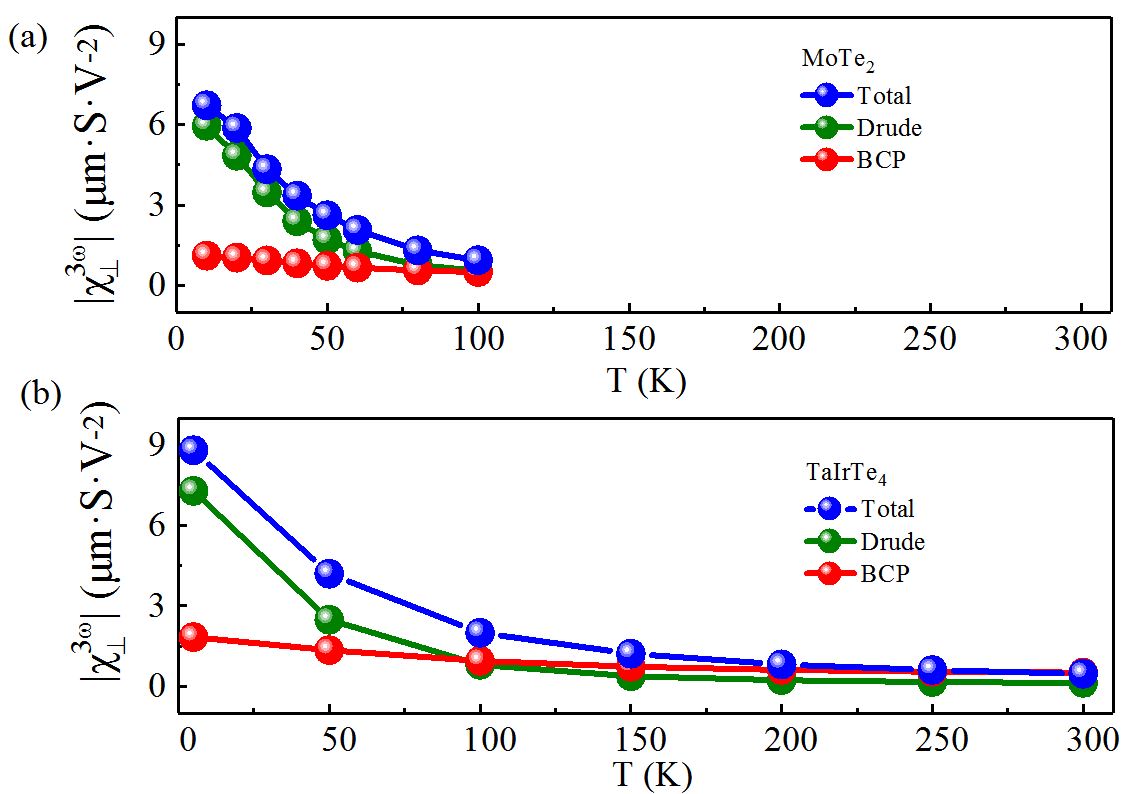


Figure S8. The contribution of the Drude-like and BCP-like part of 3^rd^ Hall effect of **(a)** MoTe_2_ in Ref. 26 and **(b)** TaIrTe_4_.

Above all, we can conclude that compared to the MoTe_2_ with smaller and faster decaying characteristics of $\chi_{\perp}^{3\omega}$ and $V_{\perp}^{3\omega}/V_{//}^{3}$, TaIrTe_4_ can host larger and slower decaying characteristics with temperature increasing. This may be the main reason for the observation of the 3^rd^ order nonlinear Hall effect in TaIrTe_4_ at room temperature.

**References**

1.Perdew, J. P.; Burke, K.; Ernzerhof, M., Generalized gradient approximation made simple. *Physical review letters* **1996,** *77* (18), 3865.

2.Grimme, S., Semiempirical GGA‐type density functional constructed with a long‐range dispersion correction. *Journal of computational chemistry* **2006,** *27* (15), 1787-1799.

3.Kerber, T.; Sierka, M.; Sauer, J., Application of semiempirical long‐range dispersion corrections to periodic systems in density functional theory. *Journal of computational chemistry* **2008,** *29* (13), 2088-2097.

4.Koepernik, K.; Kasinathan, D.; Efremov, D.; Khim, S.; Borisenko, S.; Büchner, B.; van den Brink, J., TaIrTe 4: A ternary type-II Weyl semimetal. *Physical Review B* **2016,** *93* (20), 201101.
